# Prognostic Impact of Sarcopenia on Clinical Outcomes in Malignancies Treated With Immune Checkpoint Inhibitors: A Systematic Review and Meta-Analysis

**DOI:** 10.3389/fonc.2021.726257

**Published:** 2021-08-26

**Authors:** Shuluan Li, Tianyu Wang, Gangling Tong, Xiaoyu Li, Danhui You, Minghua Cong

**Affiliations:** ^1^Department of Medical Oncology, National Cancer Center/National Clinical Research Center for Cancer/Cancer Hospital & Shenzhen Hospital, Chinese Academy of Medical Sciences and Peking Union Medical College, Shenzhen, China; ^2^Department of Breast Surgery, National Cancer Center/National Clinical Research Center for Cancer/Cancer Hospital & Shenzhen Hospital, Chinese Academy of Medical Sciences and Peking Union Medical College, Shenzhen, China; ^3^Department of Oncology, Peking University Shenzhen Hospital, Shenzhen Key Laboratory of Gastrointestinal Cancer Translational Research, Cancer Institute of Shenzhen-Peking University-Hong Kong University of Science and Technology (PKU-HKUST) Medical Center, Shenzhen, China; ^4^Department of Comprehensive Oncology, National Cancer Center/National Clinical Research Center for Cancer/Cancer Hospital, Chinese Academy of Medical Sciences and Peking Union Medical College, Beijing, China

**Keywords:** sarcopenia, malignancies, predictive factor, meta-analysis, immunotherapy

## Abstract

**Background:**

The effect of sarcopenia on the clinical outcomes of patients with malignant neoplasms receiving immune checkpoint inhibitors (ICIs) is unclear. The aim of this study was to evaluate the effect and survival of patients with malignancies and sarcopenia receiving ICIs.

**Methods:**

We systematically searched related studies in PubMed, Embase, and Cochrane Library up to March 2021 according to the inclusion and exclusion criteria. Information pertaining to the hazard ratio (HR) corresponding to 95% confidence interval (CI) of overall survival (OS) and progression-free survival (PFS) as determined by univariate and multivariate analyses; the odds ratio (OR) corresponding to the 95% CI of the disease control rate (DCR) and objective response rate (ORR); and immune-related adverse events (irAEs) was collected and analyzed using the RevMan 5.4 software. Study heterogeneity and sensitivity were also assessed.

**Results:**

A total of 19 studies were finalized that included 1763patients with lung, gastrointestinal, and head and neck cancers as well as those with melanoma, renal cell carcinoma, urothelial carcinoma, pancreatic cancer, and soft tissue sarcoma. According to univariate and multivariate analyses, patients with sarcopenia at pre-immunotherapy had poorer PFS and OS than those without. HRs and the corresponding 95% CI of PFS were 1.91(1.55–2.34, p <0.00001) and 1.46 (1.20–1.78, p =0.0001), respectively, and HRs and the corresponding 95% CI of OS were 1.78 (1.47–2.14, p <0.00001) and 1.73 (1.36–2.19, p <0.0001), respectively. Patients with sarcopenia showed poor PFS and OS during treatment. In addition, patients with sarcopenia had worse ORR (OR 0.46, 95% CI 0.28–0.74, p = 0.001) and DCR (OR 0.44, 95% CI 0.31–0.64, p<0.0001); however, the incidence of irAEs of any grade and high-grade in patients with sarcopenia did not increase, OR and the corresponding 95% CI were 0.58(0.30–1.12, p = 0.10) and 0.46(0.19–1.09, p = 0.08). Further, we performed subgroup analysis, skeletal muscle mass index (SMI) and psoas muscle mass index (PMI) stratification. In the SMI group, patients with sarcopenia had poor ORR, DCR, PFS, and OS than those without. In the PMI group, sarcopenia had poor ORR,DCR, and was a poor prognostic factor for PFS and OS according to univariate analysis but had no effect on PFS and OS according to multivariate analysis.

**Conclusions:**

Patients with malignancies and sarcopenia at pre-immunotherapy or follow-up visits had poorer clinical outcomes than those without, and sarcopenia was a poor predictive factor of ICI immunotherapy outcomes.

## Introduction

Immune checkpoint inhibitors (ICIs) are currently widely used for treating patients with solid tumors, especially non-small cell lung cancer, and melanoma and have achieved revolutionary treatment effects and long-term survival times compared with conventional chemotherapy (1, 2). ICIs inhibit tumor growth or kill tumor cells by stimulating and enhancing antitumor responses (3). However, the immunotherapeutic efficacy of ICIs is not completely satisfactory because most patients cannot benefit from immunotherapy and immune tolerance may be the main reason behind this lack of sufficient efficacy (4). Several studies have attempted to determine the type of patients who are most likely to benefit from ICI treatment. Although the expression of *PD-L1*, a mismatch repair protein, and microsatellite instability tests have helped in screening people who can benefit clinically from this treatment, the number of better indictors for accurately predicting immunotherapy outcome is insufficient (5, 6).

Sarcopenia is characterized by loss of skeletal muscle mass and function, and is a poor prognostic factor for outcomes in patients who have received chemotherapy or undergone surgery (7, 8). The prevalence of sarcopenia is high in cancer patients (9). The reasons for the high incidence of sarcopenia in patients with cancer are as follows: malnutrition, physical inactivity, inflammatory response, alterations in the hormonal milieu and imbalance of metabolism presence, and increased protein consumption (10, 11). Some studies have reported that sarcopenia in cancer patients who have not received treatment is related to postoperative complications, chemotherapy-induced toxicity, and poor survival rate (12–15).

Recently, several studies have shown the poor effect of sarcopenia on patients including lung cancer, melanoma, gastrointestinal system, urothelial carcinoma receiving ICI immunotherapy (16–18). However, whether sarcopenia is a predictive factor for clinical outcomes in patients with malignancies receiving ICIs is unclear. To date, only one meta-analysis has reported the relationship between sarcopenia and immunotherapy, mainly focusing on non-small cell lung cancer (19). Therefore, we performed this systematic review and meta-analysis to investigate the effect of sarcopenia on the efficacy of immunotherapy for different types of tumors.

## Methods

This meta-analysis was performed according to the Preferred Reporting Items for Systematic Reviews and Meta-Analyses guidelines (20).

### Search Strategy

We systematically searched the related studies in PubMed, Embase, and Cochrane Library databases from inception until July 2021. These search terms are provided in the **Supplementary Material**. Moreover, we screened the collected reference lists to identify relevant studies.

### Inclusion Criteria

The inclusion criteria for eligible studies were as follows: (1) Patients: patients diagnosed with metastatic or recurrent malignant solid tumors; (2) Intervention methods: all patients received ICI immunotherapy, including PD-1 or PD-L1 inhibitors or CTLA-4 inhibitors as first-line, second-line, or multiple lines of treatment; (3) Comparison factor: patients with sarcopenia compared with those without and sarcopenia was evaluated by computed tomography (CT) scan before immunotherapy was administered. Measurement values of the cross-sectional area of the total skeletal muscle index (SMI) or psoas muscle index (PMI) on abdominal CT at the level of the third lumbar vertebra (L3) were used to evaluate sarcopenia. Sarcopenia was defined by a definite cut-off value. (4) Outcome: The clinical outcomes of eligible studies included progression-free survival (PFS) or overall survival (OS) with reporting hazard ratio (HR) and 95% confidence interval (CI), and objective response rate (ORR), disease control rate (DCR), and immune-related adverse events (irAEs) reporting the event number of patients; and (5) Study design: randomized controlled clinical trials and observational research. Only those studies with full-text availability were selected.

### Exclusion Criteria

The exclusion criteria were as follows: Patients with benign cancers and those with malignancies but who did not receive ICI immunotherapy but received chemotherapy or underwent surgery; sarcopenia was not diagnosed by CT scan and the study had no pre-immunotherapy data and no definitive cut-off value for sarcopenia; there was no available data on OS, PFS, ORR, or DCR and no control group (non-sarcopenia); and the study type was review, case report, letter, comment, or meta-analysis, and the full text could not be accessed for such studies.

### Data Extraction and Quality Assessment

Two authors (SL and TW) independently searched and scanned the identified titles and abstracts to find relevant studies. If an abstract was identified as a potential inclusion, we screened the full text of the publication. Discrepancies were resolved by discussion. Studies were excluded on the basis of the consensus of three authors (SL, TW, and GT). We collected the following data from the included studies: first author name, publication country, publication year, sample size, patient age, tumor stage, ICI drug, sarcopenia evaluation method and cut-off value, numbers of patients with and without sarcopenia, PFS and OS corresponding HRs and 95% CIs, and irAEs. The quality of eligible studies was assessed according to the Newcastle–Ottawa Scale (NOS) criteria.

### Statistical Analysis

We used the RevMan 5.4 software for all the analyses. The effect of pre-immunotherapy sarcopenia or change related to it during treatment on PFS and OS was evaluated using HR and corresponding 95% CI values and the relationship between pre-immunotherapy sarcopenia and ORR, DCR, and irAEs was evaluated according to odds risks and the corresponding 95% CIs. Heterogeneity among studies was evaluated using I^2^ statistics and the Cochran Q test when heterogeneity was significant (I^2^ ≥50% and/or P for Q test ≤0.10), and χ^2^ <0.10 was used to define statistically significant heterogeneity. Further, subgroup analysis was performed and a fixed-effect model was used. If heterogeneity was significant, a random-effect model was used. Sensitivity analysis was performed using the leave-one-out method (sequentially removing one study each time). A funnel plot was used to assess publication bias. Statistical significance was defined as a two-sided p-value of <0.05.

## Results

### Study Selection and Characteristics

**Figure 1:**
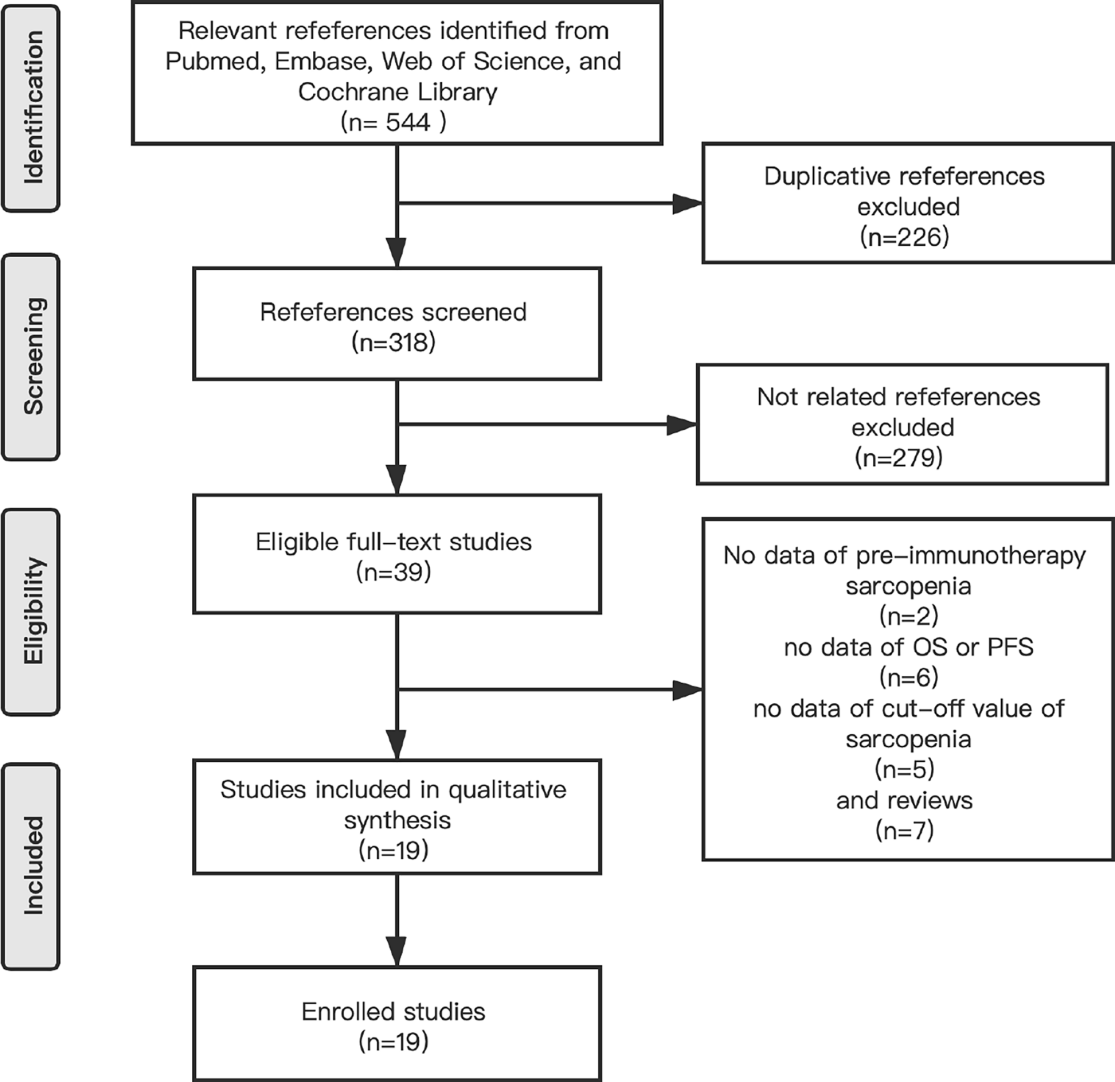
A flow chart of studies selection process.

We identified 544 references by searching for studies in PubMed, Embase, Web of Science and the Cochrane Library and 226 references were excluded because of the presence of duplicate articles. The titles and abstracts of the articles were screened and 279 references were excluded because they were irrelevant according to the inclusion criteria. Moreover, we read 39 full-text studies carefully and identified 7 reviews and 2 article without data regarding pre-immunotherapy sarcopenia, 6articles without clinical effects, and 5 articles without cut-off values for sarcopenia. Finally, 19 eligible studies were included in the meta-analysis (**Figure 1**).

These 19 studies were all retrospective in nature and included a total of 1763 patients (those with lung cancer, melanoma, renal cell carcinoma, urothelial carcinoma, gastrointestinal cancer, soft-tissue sarcoma, prostate adenocarcinoma, cervical cancer, and ICI immunotherapy contained *PD-1* or *PD-L1* inhibitors or *CTLA-4* inhibitors. In these studies, 17 studies evaluated PFS, 16 studies evaluated OS, 17 studies evaluated tumor response, and 10 studies reported irAEs of any grade. Sarcopenia was defined by measuring skeletal muscle mass using CT images. The main characteristics of the included studies are summarized in **Table 1** (17, 18, 21–37).

**Table 1:** Characteristics of the included studies.

Author/ year	Country	Sample size (M/F)	Age (years)	Patients	ICI type	Muscle mass criteria	Cut-off value	Median follow-up	Outcome	NOS score
Shiroyama/ 2019 (21)	Japan	42 (26/16)	Median sarcopenia 72 (51–87) Non-sarcopenia 69 (37–78)	Lung cancer	Nivolumab or pembrolizumab	PMI by CT at L3	Men: 6.36 cm^2^/m^2^ Women: 3.92 cm^2^/m^2^	NA	PFS, Tumor response	7
Nishioka/ 2019 (22)	Japan	38 (26/12)	Median 68.7 (46–85)	lung cancer	Nivolumab or pembrolizumab	PMA by CT at L2-L3	Change rate of PMMA,≥10%	NA	PFS, Tumor response	6
Cortellini/ 2019 (23)	Italy	23 (18/5)	Median 67 (41–82)	Lung cancer	Nivolumab	SMI by CT at L3	Men: BMI <25 kg/m^2^, 43 cm^2^/m^2^, BMI >25 kg/m^2^, 53 cm^2^/m^2^ Women: 41 cm^2^/m^2^	15.7 m	PFS, OS, Tumor response, irAEs	5
Minami/ 2020 (24)	Japan	74 (48/26)	Median (IQR): low PMI: 69 (63–74), high PMI70 (61-73)	lung cancer	Nivolumab, pembrolizumab, or atezolizumab	PMI by CT at L3	Men: 6.36 cm^2^/m^2^ Women: 3.92 cm^2^/m^2^	NA	PFS, OS, Tumor response	8
Tsukagoshi/ 2020 (25)	Japan	30 (23/7)	median 67 (47–82)	lung cancer	Nivolumab	PMI by CT at L3	Men: 6.36 cm^2^/m^2^ Women:3.92 cm^2^/m^2^	NA	PFS, OS, Tumor response, irAEs	7
Takada/ 2020 (26)	Japan	103 (84/19)	median 67 (36–88)	lung cancer	Nivolumab or pembrolizumab	SMI by CT at L3	Men: 25.63 cm^2^/m^2^ Women:21.73 cm^2^/m^2^	228 d	PFS, OS, Tumor response	6
Nishioka/ 2020 (27)	Japan	156 (101/55)	median 67 (33–85)	lung cancer	Nivolumab, pembrolizumab or atezolizumab	SMI by CT at L3	BMI <25 kg/m^2^, SMD 41 HU, SMI 43 cm^2^/m^2^ (men), 41 cm^2^/m^2^ (women), BMI ≥25 kg/m^2^, SMD 33 HU, SMI: 53 cm^2^/m^2^ (men)	2.5 m	PFS, OS, Tumor response	7
Cortellini/ 2020 (33)	Italy	100 (67/33)	median 66 (25–88)	lung cancer, melanoma, renal cell carccinoma, others	Anti-PD-1 or Anti-PD-L1	SMI by CT at L3	BMI <25 kg/m^2^: men, SMI 48.4 cm^2^/m^2^, SMD24.2HU, women: SMI: 36.9 cm^2^/ m^2^, SMD 27.9 HU BMI ≥25 kg/m^2^: men: SMI: 50.2 cm/m^2^, SMD 35.6 HU; women; SMI 59.6 cm^2^/m^2^; SMD 37.4 HU	20.3m	PFS,OS, Tumor response irAEs	7
Roch/ 2020 (28)	France	142 (93/49)	mean 63.54 ± 10.58	lung cancer	Nivolumab or pembrolizumab	SMI by CT at L3	Men: 52.4 cm^2^/m^2^ Women: 38.5 cm^2^/m^2^ Decrease ≥5% inL3-SMI during therapy	5.5m	PFS, OS, Tumor response	6
Chu/ 2020 (29)	Canada	97 (58/39)	median 56 (25–91)	melanoma	Ipilimumab	SMD by CT at L3	BMI< 25 kg/m^2^: SMD 42 HU BMI ≥25 kg/m^2^: SMD 20 HU	NA	PFS, OS Tumor response irAEs	6
Kim/ 2020 (34)	Korea	149 (93/56)	mean 57.0 ± 12.3	gastric cancer	Nivolumab, pembrolizumab	SMI by CT at L 3	Men: 49 cm^2^/m^2^ Women:31 cm^2^/m^2^	20.3m	PFS, OS, Tumor response	7
Young/ 2020 (17)	USA	287 (184/103)	median 63 (20–89) mean 61.0 ± 14.4	melanoma	Nivolumab, pembrolizumab, atezolizumab, Nivolumab + ipilimumab	SMI by CT at L3 SMD by CT at L3	BMI <25 kg/m^2^, SMI 43 cm/m^2^ (men) and 41 cm/m^2^ (women) BMI ≥26 kg/m^2^, 53 cm/m^2^ (men) and 41 cm/m^2^ (women); BMI <25 kg/m^2^, SMD 41 HU; BMI >25 kg/m^2^, SMD 33 HU.	519 d	PFS, OS, Tumor response irAEs	7
Kano/ 2020 (35)	Japan	31 (21/10)	median 70 (35–83)	gastric cancer	Nivolumab	PMI by CT at L3	Men: 3.6 cm^2^/m^2^ Women: 2.9 cm^2^/m^2^	NA	PFS, Tumor response irAEs	7
Loosen/ 2021 (30)	Germany	88 (58/30)	median 67 (34–87)	lung cancer, melanoma, urothelial carcinoma, gastrointestinal cancer, head and neck cancer, and others	Nivolumab, ipilimumab, Avelumab, Durvalumab, pembrolizumab, Nivolumab + ipilimumab	SMI and MMA by CT at L3 Δt-SMI ΔMMA	SMI: 80.09 mm^2^/cm SMD: 42.3 HU Δt-SMI: 6.18 mm^2^/cm ΔMMA:0.4HU	NA	Tumor response OS	6
Takuto /2020 (18)	Japan	27 (23/4)	median 73 (52–82)	Urothelial carcinoma	Pembrolizumab	PMI by CT at L3	Men: 6.36 cm^2^/m^2^ Women:3.92 cm^2^/m^2^	7m	PFS, OS, Tumor response irAEs	7
Fukushima/2020 (31)	Japan	28(19/9)	median 74 (70–82)	Urothelial carcinoma	Pembrolizumab	SMI by CT at L3	BMI <25 kg/m^2^, SMI 43 cm/m^2^ (men) and 41 cm/m^2^ (women) BMI ≥25 kg/m^2^, 53 cm/m^2^ (men) and 41 cm/m^2^ (women)	6m	PFS, OS, Tumor response irAEs	7
Kim&Yu /2020 (36)	Korea	102(87/15)	Median (IQR) 61.3 [54.0; 69.0]	Hepatocellular carcinoma	Nivolumab	SMI by CT at L3	Men: 42cm^2^/m^2^ Women:38 cm^2^/m^2^	21.9m	PFS, OS, Tumor response irAEs	7
Kim /2021 (37)	Korea	185(120/65)	Median 59 [51–69]	gastric cancer	Pembrolizumab Nivolumab	SMI by CT at L3	Men: 49cm^2^/m^2^ Women:31 cm^2^/m^2^	4.8m	OS, Tumor response	7
Arribas /2021 (32)	Spain	61(52/9)	median 59 (23–78) mean 57.7 ± 9.62	head and neck cancer	NA	SMI by CT at L3	SMI 42cm^2^/m^2^	9m	PFS, OS, irAEs	6

M/F, Man/Female; ICI, immune checkpoint inhibitor; NA, Not evaluated; m, month; d, day; PMI, psoas muscle mass index; SMI, skeletal muscle index; CT, computer tomography; L3, the third lumbar vertebra; BMI, body mass index; SMD, skeletal muscle fat density; PFS, progression-free survival; OS, overall survival; irAEs, immune-related adverse events.

### The Effect of Sarcopenia on Clinical Outcome

We performed a meta-analysis of the effect of sarcopenia on clinical outcomes, mainly with regard to PFS, OS, ORR, and DCR, all of which showed poor results in patients with sarcopenia than those without.

#### Progression-Free Survival (PFS) and Overall Survival (OS)

**Figure 2:**
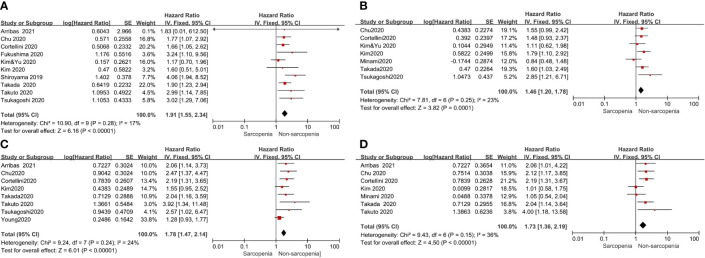
Forest plot of impact of sarcopenia on clinical efficacy. **(A)** Sarcopenia and PFS(by univariate HR), **(B)** Sarcopenia and PFS(by multivariate HR), **(C)** Sarcopenia and OS(by univariate HR), **(D)** Sarcopenia and OS(by multivariate HR).

All studies reported PFS or OS; however, a few studies were excluded to analyze PFS or OS because they did not report HR data or only reported the survival curve data. Therefore, to assess the effect of pre-immunotherapy sarcopenia on PFS, we considered 10 articles that included a total of 739 patients for univariate Cox proportional hazards regression analysis and 7 studies including a total of 655 patients for multivariate HR analysis of PFS. The meta-analyses of these studies showed that patients with sarcopenia had poorer PFS than those without; HR and the corresponding 95% CI values according to univariate analysis were 1.91 (1.55–2.34, p <0.00001) with no heterogeneity (p = 0.28, I^2^ = 17%) and 1.46(1.20–1.78, p=0.001) according to multivariate analysis with low heterogeneity (p = 0.25, I^2^ = 23%), respectively (**Figure 2**).

To determine the effect of pre-immunotherapy sarcopenia on OS, 8 studies that included a total of 860 patients were selected for univariate analysis and 7 studies that included a total of 611 patients were selected for multivariate HR analysis of OS. The meta-analyses of these studies showed that patients with sarcopenia had poorer OS. HR and the corresponding 95% CI according to univariate analysis was 1.78(1.47–2.14, p <0.00001) with low heterogeneity (p = 0.24, I^2^ = 24%), and 1.73 (1.36–2.19, p <0.00001) by multivariate analysis with moderate heterogeneity (p = 0.15, I^2^ = 36%) (**Figure 2**), respectively. Considering the low or moderate heterogeneity of the above results, a random-effect model was used and similar results were obtained.

**Figure 3:**
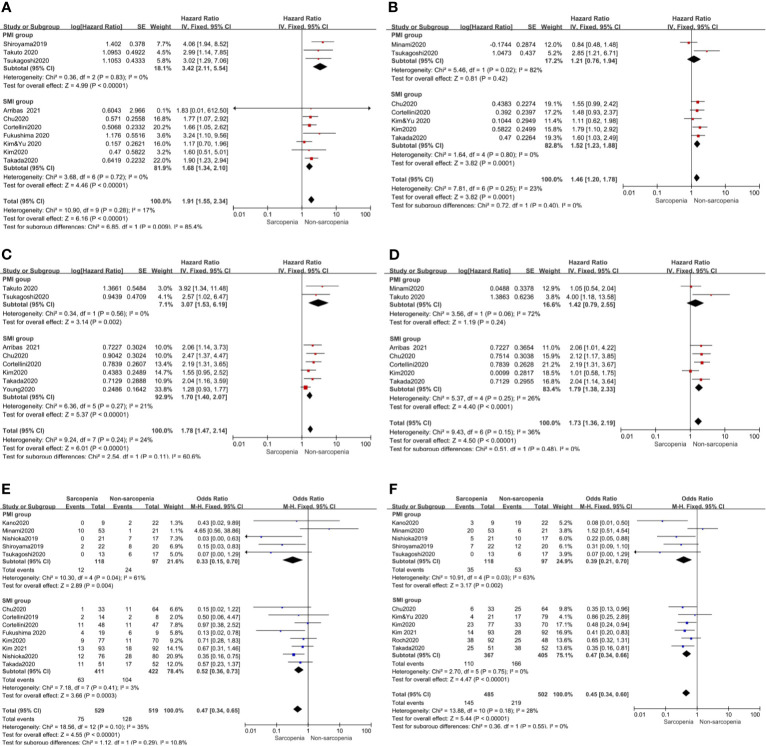
Subgroup analysis including SMI and PMI group. **(A)** Sarcopenia and PFS(by univariate HR), **(B)** Sarcopenia and PFS(by multivariate HR), **(C)** Sarcopenia and OS(by univariate HR), **(D)** Sarcopenia and OS(by multivariate HR) **(E)** Sarcopenia and ORR, **(F)** Sarcopenia and DCR.

Further, we performed subgroup analysis of SMI and PMI subgroups and determined the effect of sarcopenia on clinical outcomes. The results showed that in the SMI group, sarcopenia was a poor prognostic factor for PFS and OS in univariate and multivariate analyses. However, in the PMI group, sarcopenia was a poor prognostic factor for PFS and OS in univariate analysis but had no effect on PFS and OS in multivariate analysis (**Figure 3**).

One study evaluated the effect of developing sarcopenia during treatment on PFS and OS. The results of this study showed that patients with developing sarcopenia had worse PFS and OS than those without change. The HR (95% CI) was 2.45 (1.09–5.53) and 3.87 (1.60–9.34) respectively (28). Another study evaluated the effect of increasing or decreasing L3SMI/median skeletal muscle attenuation and delta (∆) L3SMI (∆L3SMI) three months after ICI therapy on OS and reported that ∆L3SMI became a prognostic factor for OS with HR values (95% CI) of 0.929 (0.898–0.960) and 0.925 (0.890–0.961) according to univariate and univariate Cox-regression analysis, respectively (30).

#### Objective Response Rate (ORR) and Disease Control Rate (DCR)

**Figure 4:**
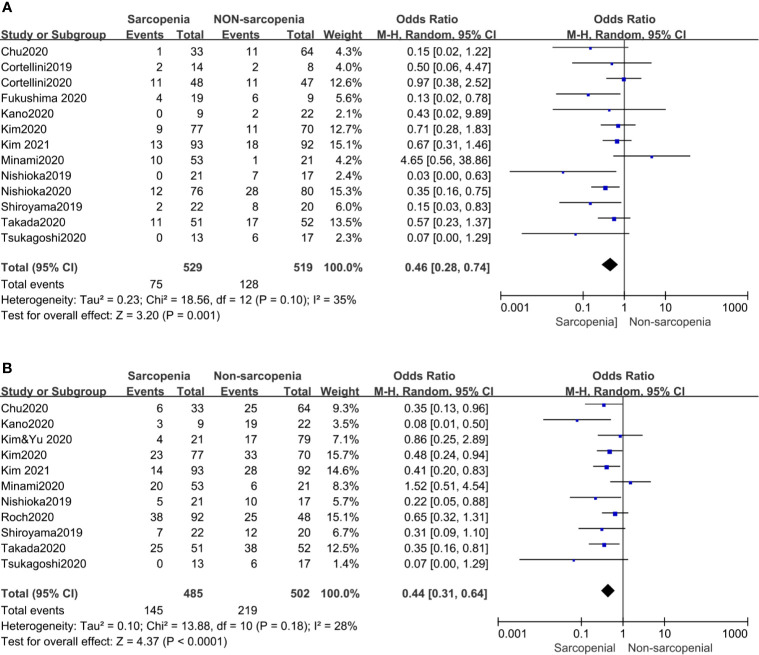
Forest plot of impact of sarcopenia on tumor response. **(A)** Sarcopenia and ORR, **(B)** Sarcopenia and DCR.

Our meta-analysis showed that patients with sarcopenia had worse ORR (OR 0.46, 95% CI 0.28–0.74, p = 0.001) and DCR (OR 0.44, 95% CI 0.31–0.64, p <0.0001) compared to those without sarcopenia. **Figure 4** shows all the low inconsistencies corresponding to the I^2^ value (p = 0.10, I^2^ = 35% and p = 0.18, I^2^ = 28%, respectively) using the fixed-effect model. A random-effect model was then used to analyze the same parameters and similar results were obtained. Patients with sarcopenia in both SMI and PMI subgroups had poor ORR and DCR (**Figure 3**).

#### Immune-Related Adverse Events (irAEs)

**Figure 5:**
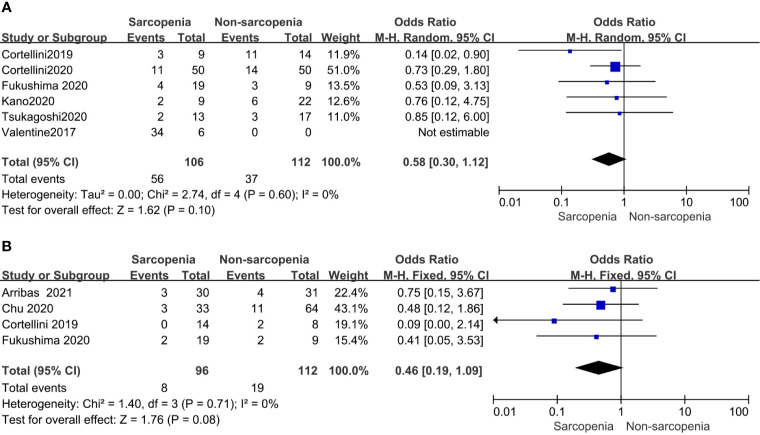
Forest plot of impact of sarcopenia on iAEs **(A)**. Sarcopenia and any grade iAEs, **(B)** Sarcopenia and high grade iAEs.

Of the 19 included studies, 10 articles reported irAEs, of which 5articles of any grade iAEs and 4 studies of high grade iAEs were suitable for analysis. And found that in patients with sarcopenia, the incidence rate of irAEs neither of any grade nor high grade increased compared to those without sarcopenia. OR and the corresponding 95% CI were 0.58(0.30–1.12, p = 0.10) and 0.46(0.19–1.09, p = 0.08), respectively. and both showed no heterogeneity with p(0.60, 0.63) and I^2^ (0% and 0%), respectively (**Figure 5**).

### Assessment of Quality and Risk of Bias in Included Studies and Publication Bias

**Figure 6:**
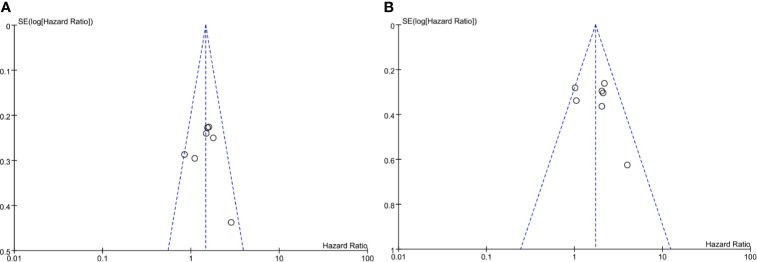
Funel plot of impact of publication bias. **(A)** Sarcopenia and PFS (by multivariate HR), **(B)** Sarcopenia and OS (by multivariate HR).

The included articles were all retrospective studies, so the quality assessment and risk of bias of analysis were performed using the 9-point Newcastle–Ottawa (NOS) scale. The evaluation results are shown in **Table 1**. Publication bias related to PFS and OS determined using a funnel plot showed no significant publication bias (**Figure 6**).

## Discussion

Immunotherapy has been gradually and widely used for the treatment of various types of cancers (1, 2). Compared with traditional chemotherapy, patients with advanced solid malignancies receiving immunotherapy have a lower risk of developing adverse events and the proportion of patients with serious adverse reactions above grade III is significantly lower (16.5% *vs.* 41.09%) (38). Sarcopenia is gradually attracting attention as a factor affecting patient prognosis. Sarcopenia is defined as age-associated loss of skeletal muscle mass and function. The incidence of sarcopenia in patients with cancer is high: it is 43% in patients with non-small cell lung cancer (NSCLC), 52% in patients with small cell lung cancer (SCLC) (39), 57% in patients with gastric cancer (40), and 29% in patients with metastatic renal cell carcinoma (41). There are some causes of sarcopenia during chemotherapy, targeted therapy, and immunotherapy. Vitamin D suppresses the expression and activity of *FOXO1*, an atrophy-related gene, commonly observed during conditions such as malnutrition and cancer (42, 43). Increased levels of tumor necrosis factor (TNF-α), interferon (IFN)-γ, and interleukin (IL)-6 are associated with increased muscle catabolism and reduced muscle protein synthesis (44).

Sarcopenia is believed to be associated with poor outcomes in patients with solid tumors who have received chemotherapy or have undergone surgery. It is also related to increased chemotherapeutic toxicity and postoperative complications (45–47). However, the effect of sarcopenia on immunotherapy outcome remains unclear. A previous study showed that sarcopenia affected the immune system leading to immune senescence (48). Hence, sarcopenia was considered to have a deleterious effect on the antitumor response by immunotherapy. Most studies have reported that sarcopenia is associated with worse treatment response and shorter survival in patients with NSCLC treated with ICI immunotherapy (19). This analysis was performed mainly on lung cancer data and did not include other cancer types. Other studies also showed that sarcopenia was the negative impact on clinical outcomes in melanoma, urothelial carcinoma patients (18, 29, 30). In addition, some studies (23, 28) have reported that sarcopenia had no effect on the outcome of malignancies. Therefore, for the first time, we performed a meta-analysis of the effect of sarcopenia on various tumors. Furthermore, we verified that sarcopenia affects the efficacy of ICIs administered for treating various types of tumors including lung cancer, melanoma, renal cell carcinoma, urothelial carcinoma, gastrointestinal cancer, soft-tissue sarcoma, prostate adenocarcinoma, and cervical cancer. In our analysis, patients with sarcopenia had the worst ORR and DCR values. Both univariate and multivariate analyses of HR for PFS and OS showed that patients with sarcopenia had poorer survival than those without sarcopenia and sarcopenia was an independent prognostic factor. Moreover, sarcopenia developing during treatment *versus* no changes related to skeletal muscle mass during treatment also affected patient survival. Only a few studies have reported the effect of sarcopenia on irAEs, which showed that sarcopenia increased their incidence (49, 50). However, a meta-analysis reported that sarcopenia had no relation with irAEs (19). Our analysis also showed that sarcopenia did not increase the incidence of irAEs. Due to the small number of studies, more data are needed to confirm this.

Sarcopenia can be diagnosed by dual-energy X-ray absorptiometry scan, bioelectrical impedance analysis, CT, and magnetic resonance imaging (MRI) (51). Measurements of the cross-sectional area of total skeletal muscles on abdominal CT at the level of the third lumbar vertebra (L3) and determining the total SMI are typically used to evaluate sarcopenia in clinical practice, which is considered the gold standard method (52, 53). The muscles evaluated by the above methods include the psoas, erector spinae, quadratus lumborum, transversus abdominis, external obliques, internal obliques, and rectus abdominis, which are considered to represent whole-body muscles. However, measuring the mass of these muscles is cumbersome, and hence, a previous study determined the psoas muscle index (PMI) by measuring the cross-section of this muscle at the L3 level to evaluate muscle mass (54). However, a previous study reported that the psoas major cannot represent whole-body skeletal muscle mass (55). The criteria for determining cut-off values for sarcopenia based on CT or MRI scans are inconsistent. Different studies have reported varied values (7, 56). The evaluation indicators of sarcopenia in the studies included in our analysis were SMI or PMI measured by CT and determined cut-off values. Subgroup analysis showed that sarcopenia affected ORR and DCR in both the SMI and PMI groups. In the SMI group, sarcopenia was a poor prognostic factor for PFS and OS according to both univariate and multivariate analyses. However, in the PMI group, sarcopenia was not an independent prognostic factor for PFS and OS according to multivariate analysis. However, these results are required to be further confirmed because only two studies were included.

Both sarcopenia and the immune response of cancer patients affect each other and result in poor prognosis. Systemic inflammatory markers secrete cytokines, chemokines, and growth factors and play a key role in carcinogenesis and metastasis (57). Tumor-related skeletal muscle exhaustion is also believed to be induced by tumor-related inflammation (57). Previous studies have shown that sarcopenia has a significant linear relationship with the levels of systemic inflammation markers (such as serum C-reactive protein) (58). Furthermore, a previous study reported that the neutrophil/lymphocyte ratio (NLR) of patients with sarcopenia was higher than that of patients without sarcopenia (59). Another study reported that in stage II colon cancer patients receiving adjuvant chemotherapy, NLR was an independent predictor of OS, and as the NLR increased, the number of lymphocytes and lymphocyte-mediated immune response against malignant tumors decreased (60). On the other hand, because muscle cells can produce anti-proliferative mediators, the reduction in muscle cells reduces the levels of these mediators, possibly contributing to cancer recurrence (61).

Our analysis strictly screened the included studies; therefore, there was no obvious publication bias or heterogeneity. Both univariate and multivariate analyses were performed to evaluate the clinical outcomes of the effect of sarcopenia on patients with different types of cancers who received ICI treatment. Moreover, according to the diagnosis of sarcopenia by SMI and PMI, we stratified and analyzed its effect on survival in the PMI and SMI subgroups. However, our meta-analysis has several limitations. First, we included only retrospective studies, so the results could be affected by specific biases in the initial study. Second, the methods for assessing sarcopenia were different, and the cut-off values for diagnosing sarcopenia were inconsistent among the included studies. Third, although we performed subgroup analysis, considering the number of included studies and failure to evaluate the prognosis of sarcopenia and its effect on OS in the PMI group, we could not conclude whether PMI can be used to assess sarcopenia. Low muscle mass thresholds also depend on factors such as age, race, and region of the studied population. Finally, most of the studies included in our meta-analysis were performed on Asian patients. Our findings need to be confirmed by performing further studies on patients from other regions.

Based on our results, we concluded that sarcopenia is a poor prognostic factor for cancer patients who received ICI treatment. Compared with non-sarcopenia patients, patients with sarcopenia had a lower treatment response and shorter OS. Therefore, when immunotherapy is administered, we suggest that the effect of sarcopenia on the therapeutic effect of the treatment should be first known. During the follow-up period, CT is recommended to evaluate muscle mass while evaluating the therapeutic effect of immunotherapy.

## Data Availability Statement

The original contributions presented in the study are included in the article/**Supplementary Material**, further inquiries can be directed to the corresponding author.

## Author Contributions 

All authors listed have made a substantial, direct, and intellectual contribution to the work and approved it for publication.

## Funding

This work was supported by the Shenzhen Key Medical Discipline Construction Fund (No.Sn 320ZXK095) and Wu Jieping Medical Foundation (6750.17536).

## Conflict of Interest

The authors declare that the research was conducted in the absence of any commercial or financial relationships that could be construed as a potential conflict of interest.

## Publisher’s Note

All claims expressed in this article are solely those of the authors and do not necessarily represent those of their affiliated organizations, or those of the publisher, the editors and the reviewers. Any product that may be evaluated in this article, or claim that may be made by its manufacturer, is not guaranteed or endorsed by the publisher.

## References

[1] Doroshow DB Sanmamed MF Hastings K Politi K Rimm DL Chen L. Immunotherapy in Non-Small Cell Lung Cancer: Facts and Hopes. Clin Cancer Res (2019) 25:4592–602. 10.1158/1078-0432.Ccr-18-1538 PMC6679805 30824587

[2] Wolchok JD Hodi FS Weber JS Allison JP Urba WJ Robert C. Development of Ipilimumab: A Novel Immunotherapeutic Approach for the Treatment of Advanced Melanoma. Ann N Y Acad Sci (2013) 1291:1–13. 10.1111/nyas.12180 23772560 PMC3910157

[3] Cao J Yan Q. Cancer Epigenetics, Tumor Immunity, and Immunotherapy. Trends Cancer (2020) 6:580–92. 10.1016/j.trecan.2020.02.003 PMC7330177 32610068

[4] Wei Y Du Q Jiang X Li L Li T Li M. Efficacy and Safety of Combination Immunotherapy for Malignant Solid Tumors: A Systematic Review and Meta-Analysis. Crit Rev Oncol Hematol (2019) 138:178–89. 10.1016/j.critrevonc.2019.04.008 31092375

[5] Gibney GT Weiner LM Atkins MB. Predictive Biomarkers for Checkpoint Inhibitor-Based Immunotherapy. Lancet Oncol (2016) 17:e542–51. 10.1016/s1470-2045(16)30406-5 PMC5702534 27924752

[6] Walk EE Yohe SL Beckman A Schade A Zutter MM Pfeifer J. The Cancer Immunotherapy Biomarker Testing Landscape. Arch Pathol Lab Med (2020) 144:706–24. 10.5858/arpa.2018-0584-CP 31714809

[7] Martin L Birdsell L Macdonald N Reiman T Clandinin MT McCargar LJ. Cancer Cachexia in the Age of Obesity: Skeletal Muscle Depletion is a Powerful Prognostic Factor, Independent of Body Mass Index. J Clin Oncol (2013) 31:1539–47. 10.1200/jco.2012.45.2722 23530101

[8] Shachar SS Williams GR Muss HB Nishijima TF. Prognostic Value of Sarcopenia in Adults With Solid Tumours: A Meta-Analysis and Systematic Review. Eur J Cancer (2016) 57:58–67. 10.1016/j.ejca.2015.12.030 26882087

[9] Cruz-Jentoft AJ Landi F Schneider SM Zúñiga C Arai H Boirie Y. Prevalence of and Interventions for Sarcopenia in Ageing Adults: A Systematic Review. Report of the International Sarcopenia Initiative (EWGSOP and IWGS). Age Ageing (2014) 43:748–59. 10.1093/ageing/afu115 PMC4204661 25241753

[10] Bilen MA Martini DJ Liu Y Shabto JM Brown JT Williams M. Combined Effect of Sarcopenia and Systemic Inflammation on Survival in Patients With Advanced Stage Cancer Treated With Immunotherapy. Oncologist (2020) 25:e528–35. 10.1634/theoncologist.2019-0751 PMC7066707 32162807

[11] Argilés JM Busquets S Felipe A López-Soriano FJ. Molecular Mechanisms Involved in Muscle Wasting in Cancer and Ageing: Cachexia *Versus* Sarcopenia. Int J Biochem Cell Biol (2005) 37:1084–104. 10.1016/j.biocel.2004.10.003 15743680

[12] Cousin S Hollebecque A Koscielny S Mir O Varga A Baracos VE. Low Skeletal Muscle is Associated With Toxicity in Patients Included in Phase I Trials. Invest New Drugs (2014) 32:382–7. 10.1007/s10637-013-0053-6 24343673

[13] Buettner S Wagner D Kim Y Margonis GA Makary MA Wilson A. Inclusion of Sarcopenia Outperforms the Modified Frailty Index in Predicting 1-Year Mortality Among 1,326 Patients Undergoing Gastrointestinal Surgery for a Malignant Indication. J Am Coll Surg (2016) 222:397–407.e392. 10.1016/j.jamcollsurg.2015.12.020 26803743

[14] Shachar SS Deal AM Weinberg M Williams GR Nyrop KA Popuri K. Body Composition as a Predictor of Toxicity in Patients Receiving Anthracycline and Taxane-Based Chemotherapy for Early-Stage Breast Cancer. Clin Cancer Res (2017) 23:3537–43. 10.1158/1078-0432.Ccr-16-2266 PMC5511549 28143874

[15] Nishigori T Okabe H Tanaka E Tsunoda S Hisamori S Sakai Y. Sarcopenia as a Predictor of Pulmonary Complications After Esophagectomy for Thoracic Esophageal Cancer. J Surg Oncol (2016) 113:678–84. 10.1002/jso.24214 26936808

[16] Khaddour K Gomez-Perez SL Jain N Patel JD Boumber Y. Obesity, Sarcopenia, and Outcomes in Non-Small Cell Lung Cancer Patients Treated With Immune Checkpoint Inhibitors and Tyrosine Kinase Inhibitors. Front Oncol (2020) 10:576314. 10.3389/fonc.2020.576314 33194687 PMC7607047

[17] Young AC Quach HT Song H Davis EJ Moslehi JJ Ye F. Impact of Body Composition on Outcomes From Anti-PD1 +/- Anti-CTLA-4 Treatment in Melanoma. J Immunother Cancer (2020) 8(2):e000821. 10.1136/jitc-2020-000821 32747470 PMC7398101

[18] Shimizu T Miyake M Hori S Ichikawa K Omori C Iemura Y. Clinical Impact of Sarcopenia and Inflammatory/Nutritional Markers in Patients With Unresectable Metastatic Urothelial Carcinoma Treated With Pembrolizumab. Diagnostics (Basel) (2020) 10(5):310. 10.3390/diagnostics10050310 PMC7277993 32429323

[19] Wang J Cao L Xu S. Sarcopenia Affects Clinical Efficacy of Immune Checkpoint Inhibitors in non-Small Cell Lung Cancer Patients: A Systematic Review and Meta-Analysis. Int Immunopharmacol (2020) 88:106907. 10.1016/j.intimp.2020.106907 33182031

[20] Moher D Liberati A Tetzlaff J Altman DG. Preferred Reporting Items for Systematic Reviews and Meta-Analyses: The PRISMA Statement. PloS Med (2009) 6:e1000097. 10.1371/journal.pmed.1000097 19621072 PMC2707599

[21] Shiroyama T Nagatomo I Koyama S Hirata H Nishida S Miyake K. Impact of Sarcopenia in Patients With Advanced Non-Small Cell Lung Cancer Treated With PD-1 Inhibitors: A Preliminary Retrospective Study. Sci Rep (2019) 9:2447. 10.1038/s41598-019-39120-6 30792455 PMC6385253

[22] Nishioka N Uchino J Hirai S Katayama Y Yoshimura A Okura N. Association of Sarcopenia With and Efficacy of Anti-PD-1/PD-L1 Therapy in Non-Small-Cell Lung Cancer. J Clin Med (2019) 8(4):450. 10.3390/jcm8040450 PMC6518257 30987236

[23] Cortellini A Verna L Porzio G Bozzetti F Palumbo P Masciocchi C. Predictive Value of Skeletal Muscle Mass for Immunotherapy With Nivolumab in non-Small Cell Lung Cancer Patients: A “Hypothesis-Generator” Preliminary Report. Thorac Cancer (2019) 10:347–51. 10.1111/1759-7714.12965 PMC6360197 30600905

[24] Minami S Ihara S Tanaka T Komuta K. Sarcopenia and Visceral Adiposity Did Not Affect Efficacy of Immune-Checkpoint Inhibitor Monotherapy for Pretreated Patients With Advanced Non-Small Cell Lung Cancer. World J Oncol (2020) 11:9–22. 10.14740/wjon1225 32095185 PMC7011908

[25] Tsukagoshi M Yokobori T Yajima T Maeno T Shimizu K Mogi A. Skeletal Muscle Mass Predicts the Outcome of Nivolumab Treatment for Non-Small Cell Lung Cancer. Med (Baltimore) (2020) 99:e19059. 10.1097/md.0000000000019059 PMC7035054 32049805

[26] Takada K Yoneshima Y Tanaka K Okamoto I Shimokawa M Wakasu S. Clinical Impact of Skeletal Muscle Area in Patients With Non-Small Cell Lung Cancer Treated With Anti-PD-1 Inhibitors. J Cancer Res Clin Oncol (2020) 146:1217–25. 10.1007/s00432-020-03146-5 PMC11804506 32025867

[27] Nishioka N Naito T Notsu A Mori K Kodama H Miyawaki E. Unfavorable Impact of Decreased Muscle Quality on the Efficacy of Immunotherapy for Advanced Non-Small Cell Lung Cancer. Cancer Med (2021) 10:247–56. 10.1002/cam4.3631 PMC7826480 33300678

[28] Roch B Coffy A Jean-Baptiste S Palaysi E Daures JP Pujol JL. Cachexia - Sarcopenia as a Determinant of Disease Control Rate and Survival in non-Small Lung Cancer Patients Receiving Immune-Checkpoint Inhibitors. Lung Cancer (2020) 143:19–26. 10.1016/j.lungcan.2020.03.003 32200137

[29] Chu MP Li Y Ghosh S Sass S Smylie M Walker J. Body Composition is Prognostic and Predictive of Ipilimumab Activity in Metastatic Melanoma. J Cachexia Sarcopenia Muscle (2020) 11:748–55. 10.1002/jcsm.12538 PMC7296257 32053287

[30] Loosen SH van den Bosch V Gorgulho J Schulze-Hagen M Kandler J Jördens MS. Progressive Sarcopenia Correlates With Poor Response and Outcome to Immune Checkpoint Inhibitor Therapy. J Clin Med (2021) 10(7):1361. 10.3390/jcm10071361 33806224 PMC8036296

[31] Fukushima H Fukuda S Moriyama S Uehara S Yasuda Y Tanaka H. Impact of Sarcopenia on the Efficacy of Pembrolizumab in Patients With Advanced Urothelial Carcinoma: A Preliminary Report. Anticancer Drugs (2020) 31:866–71. 10.1097/cad.0000000000000982 32740015

[32] Arribas L Plana M Taberna M Sospedra M Vilariño N Oliva M. Predictive Value of Skeletal Muscle Mass in Recurrent/Metastatic Head and Neck Squamous Cell Carcinoma Patients Treated With Immune Checkpoint Inhibitors. Front Oncol (2021) 11:699668. 10.3389/fonc.2021.699668 34249760 PMC8267860

[33] Cortellini A Bozzetti F Palumbo P Brocco D Di Marino P Tinari N. Weighing the Role of Skeletal Muscle Mass and Muscle Density in Cancer Patients Receiving PD-1/PD-L1 Checkpoint Inhibitors: A Multicenter Real-Life Study. Sci Rep (2020) 10:1456. 10.1038/s41598-020-58498-2 31996766 PMC6989679

[34] Kim YY Lee J Jeong WK Kim ST Kim JH Hong JY. Prognostic Significance of Sarcopenia in Microsatellite-Stable Gastric Cancer Patients Treated With Programmed Death-1 Inhibitors. Gastric Cancer (2021) 24:457–66. 10.1007/s10120-020-01124-x 32970267

[35] Kano M Hihara J Tokumoto N Kohashi T Hara T Shimbara K. Association Between Skeletal Muscle Loss and the Response to Nivolumab Immunotherapy in Advanced Gastric Cancer Patients. Int J Clin Oncol (2021) 26:523–31. 10.1007/s10147-020-01833-4 33226523

[36] Kim N Yu JI Park HC Yoo GS Choi C Hong JY. Incorporating Sarcopenia and Inflammation With Radiation Therapy in Patients With Hepatocellular Carcinoma Treated With Nivolumab. Cancer Immunol Immunother (2021) 70:1593–603. 10.1007/s00262-020-02794-3 PMC10991354 33231725

[37] Kim N Yu JI Lim DH Lee J Kim ST Hong JY. Prognostic Impact of Sarcopenia and Radiotherapy in Patients With Advanced Gastric Cancer Treated With Anti-PD-1 Antibody. Front Immunol (2021) 12:701668. 10.3389/fimmu.2021.701668 34305941 PMC8298191

[38] Magee DE Hird AE Klaassen Z Sridhar SS Nam RK Wallis CJD. Adverse Event Profile for Immunotherapy Agents Compared With Chemotherapy in Solid Organ Tumors: A Systematic Review and Meta-Analysis of Randomized Clinical Trials. Ann Oncol (2020) 31:50–60. 10.1016/j.annonc.2019.10.008 31912796

[39] Yang M Shen Y Tan L Li W. Prognostic Value of Sarcopenia in Lung Cancer: A Systematic Review and Meta-Analysis. Chest (2019) 156:101–11. 10.1016/j.chest.2019.04.115 31128115

[40] Tegels JJ van Vugt JL Reisinger KW Hulsewé KW Hoofwijk AG Derikx JP. Sarcopenia is Highly Prevalent in Patients Undergoing Surgery for Gastric Cancer But Not Associated With Worse Outcomes. J Surg Oncol (2015) 112:403–7. 10.1002/jso.24015 26331988

[41] Sharma P Zargar-Shoshtari K Caracciolo JT Fishman M Poch MA Pow-Sang J. Sarcopenia as a Predictor of Overall Survival After Cytoreductive Nephrectomy for Metastatic Renal Cell Carcinoma. Urol Oncol (2015) 33:339.e317–323. 10.1016/j.urolonc.2015.01.011 26094169

[42] Uchitomi R Oyabu M Kamei Y. Vitamin D and Sarcopenia: Potential of Vitamin D Supplementation in Sarcopenia Prevention and Treatment. Nutrients (2020) 12(10):3189. 10.3390/nu12103189 PMC7603112 33086536

[43] Kamei Y Miura S Suzuki M Kai Y Mizukami J Taniguchi T. Skeletal Muscle FOXO1 (FKHR) Transgenic Mice Have Less Skeletal Muscle Mass, Down-Regulated Type I (Slow Twitch/Red Muscle) Fiber Genes, and Impaired Glycemic Control. J Biol Chem (2004) 279:41114–23. 10.1074/jbc.M400674200 15272020

[44] Jeejeebhoy KN. Malnutrition, Fatigue, Frailty, Vulnerability, Sarcopenia and Cachexia: Overlap of Clinical Features. Curr Opin Clin Nutr Metab Care (2012) 15:213–9. 10.1097/MCO.0b013e328352694f 22450775

[45] Rinninella E Cintoni M Raoul P Pozzo C Strippoli A Bria E. Muscle Mass, Assessed at Diagnosis by L3-CT Scan as a Prognostic Marker of Clinical Outcomes in Patients With Gastric Cancer: A Systematic Review and Meta-Analysis. Clin Nutr (2020) 39:2045–54. 10.1016/j.clnu.2019.10.021 31718876

[46] Buentzel J Heinz J Bleckmann A Bauer C Röver C Bohnenberger H. Sarcopenia as Prognostic Factor in Lung Cancer Patients: A Systematic Review and Meta-Analysis. Anticancer Res (2019) 39:4603–12. 10.21873/anticanres.13640 31519557

[47] Deng HY Hou L Zha P Huang KL Peng L. Sarcopenia is an Independent Unfavorable Prognostic Factor of non-Small Cell Lung Cancer After Surgical Resection: A Comprehensive Systematic Review and Meta-Analysis. Eur J Surg Oncol (2019) 45:728–35. 10.1016/j.ejso.2018.09.026 30348603

[48] Nelke C Dziewas R Minnerup J Meuth SG Ruck T. Skeletal Muscle as Potential Central Link Between Sarcopenia and Immune Senescence. EBioMedicine (2019) 49:381–8. 10.1016/j.ebiom.2019.10.034 PMC6945275 31662290

[49] Heidelberger V Goldwasser F Kramkimel N Jouinot A Huillard O Boudou-Rouquette P. Sarcopenic Overweight is Associated With Early Acute Limiting Toxicity of Anti-PD1 Checkpoint Inhibitors in Melanoma Patients. Invest New Drugs (2017) 35:436–41. 10.1007/s10637-017-0464-x 28396974

[50] Hirsch L Bellesoeur A Boudou-Rouquette P Arrondeau J Thomas-Schoemann A Kirchgesner J. The Impact of Body Composition Parameters on Severe Toxicity of Nivolumab. Eur J Cancer (2020) 124:170–7. 10.1016/j.ejca.2019.11.003 31794927

[51] Cruz-Jentoft AJ Baeyens JP Bauer JM Boirie Y Cederholm T Landi F. Sarcopenia: European Consensus on Definition and Diagnosis: Report of the European Working Group on Sarcopenia in Older People. Age Ageing (2010) 39:412–23. 10.1093/ageing/afq034 PMC2886201 20392703

[52] Fearon K Strasser F Anker SD Bosaeus I Bruera E Fainsinger RL. Definition and Classification of Cancer Cachexia: An International Consensus. Lancet Oncol (2011) 12:489–95. 10.1016/s1470-2045(10)70218-7 21296615

[53] Mourtzakis M Prado CM Lieffers JR Reiman T McCargar LJ Baracos VE. A Practical and Precise Approach to Quantification of Body Composition in Cancer Patients Using Computed Tomography Images Acquired During Routine Care. Appl Physiol Nutr Metab (2008) 33:997–1006. 10.1139/h08-075 18923576

[54] Gomez-Perez SL Haus JM Sheean P Patel B Mar W Chaudhry V. Measuring Abdominal Circumference and Skeletal Muscle From a Single Cross-Sectional Computed Tomography Image: A Step-By-Step Guide for Clinicians Using National Institutes of Health ImageJ. JPEN J Parenter Enteral Nutr (2016) 40:308–18. 10.1177/0148607115604149 PMC4767633 26392166

[55] Rutten IJG Ubachs J Kruitwagen R Beets-Tan RGH Olde Damink SWM Van Gorp T. Psoas Muscle Area is Not Representative of Total Skeletal Muscle Area in the Assessment of Sarcopenia in Ovarian Cancer. J Cachexia Sarcopenia Muscle (2017) 8:630–8. 10.1002/jcsm.12180 PMC5566632 28513088

[56] Prado CM Lieffers JR McCargar LJ Reiman T Sawyer MB Martin L. Prevalence and Clinical Implications of Sarcopenic Obesity in Patients With Solid Tumours of the Respiratory and Gastrointestinal Tracts: A Population-Based Study. Lancet Oncol (2008) 9:629–35. 10.1016/s1470-2045(08)70153-0 18539529

[57] Wu Y Zhou BP. Inflammation: A Driving Force Speeds Cancer Metastasis. Cell Cycle (2009) 8:3267–73. 10.4161/cc.8.20.9699 PMC3702728 19770594

[58] Kim EY Kim YS Seo JY Park I Ahn HK Jeong YM. The Relationship Between Sarcopenia and Systemic Inflammatory Response for Cancer Cachexia in Small Cell Lung Cancer. PloS One (2016) 11:e0161125. 10.1371/journal.pone.0161125 27537502 PMC4990336

[59] Tsukioka T Izumi N Kyukwang C Komatsu H Toda M Hara K. Loss of Muscle Mass is a Novel Predictor of Postoperative Early Recurrence in N2-Positive Non-Small-Cell Lung Cancer. Ann Thorac Cardiovasc Surg (2018) 24:121–6. 10.5761/atcs.oa.17-00215 PMC6033530 29459570

[60] Hung HY Chen JS Yeh CY Changchien CR Tang R Hsieh PS. Effect of Preoperative Neutrophil-Lymphocyte Ratio on the Surgical Outcomes of Stage II Colon Cancer Patients Who do Not Receive Adjuvant Chemotherapy. Int J Colorectal Dis (2011) 26:1059–65. 10.1007/s00384-011-1192-x 21479566

[61] Fishman P Bar-Yehuda S Vagman L. Adenosine and Other Low Molecular Weight Factors Released by Muscle Cells Inhibit Tumor Cell Growth. Cancer Res (1998) 58:3181–7. 9679987

