# Supplementary material for: Prognostic Impact of Sarcopenia on Clinical Outcomes in Malignancies Treated With Immune Checkpoint Inhibitors: A Systematic Review and Meta-Analysis
Source: Front Oncol. 2021 Aug 26;11:726257. doi: 10.3389/fonc.2021.726257 (PMC8427761; doi:10.3389/fonc.2021.726257)
Supplement: Supplementary file 1 [file DataSheet_1.docx]

**Search strategy：**

The search terms were conducted as follows:

“neoplasia, ” “neoplasias,” “neoplasm,” “tumors,” “tumor,” “cancer,” “cancers,” “malignancy,” “malignancies,” “malignant neoplasms,” “malignant Neoplasm,” “neoplasm malignant,” “neoplasms malignant,” “benign neoplasms,” “neoplasms, benign,” “benign neoplasm,” “Neoplasm, Benign,”“Carcinoma,”“Carcinomas,”“Epithelial Neoplasms, Malignant,”“Malignant Epithelial Tumor”.

“checkpoint Inhibitors, Immune,” “immune Checkpoint Inhibitor,” “checkpoint Inhibitor, Immune,” “immune checkpoint blockers,” “Checkpoint Blockers, Immune,” “Immune Checkpoint Blockade,” “Checkpoint Blockade, Immune,” “Immune Checkpoint Inhibition,” “Checkpoint Inhibition, Immune,” “PD-L1 Inhibitors,” “PD L1 Inhibitors,” “PD-L1 Inhibitor,” “PD L1 Inhibitor,” “Programmed Death-Ligand 1 Inhibitors,” “Programmed Death Ligand 1 Inhibitors,” “CTLA-4 Inhibitors,” “CTLA 4 Inhibitors,” “CTLA-4 Inhibitor,” “CTLA 4 Inhibitor,” “T-Lymphocyte-Associated Protein 4 Inhibitors,” “Cytotoxic T Lymphocyte Associated Protein 4 Inhibitors,” “Cytotoxic T-Lymphocyte-Associated Protein 4 Inhibitor,” “Cytotoxic T Lymphocyte Associated Protein 4 Inhibitor,” “PD-1 Inhibitors,” “PD 1 Inhibitors,” “PD-1 Inhibitor,” “Inhibitor, PD-1,” “Programmed Cell Death Protein 1 Inhibitor,” “Programmed Cell Death Protein 1 Inhibitors,” “PD-1-PD-L1 Blockade,” “Blockade, PD-1-PD-L1,” “PD 1 PD L1 Blockade,”“pembrolizumab,”“nivolumab,”“atezolizumab,”“ipilimumab,”“avelumab,”“tremelimumab,”“durvalumab.”.

“Sarcopenia,” “Myopenia,” “Muscle atrophy,” “Muscle wasting,” “Muscle attenuation,” “Muscle deletion,” “Muscle loss,” “Muscle weak,” “Muscle reduction,” “Muscle strength,” “Muscle mass,” “Muscular atrophy,” “Muscular wasting,” “Muscular attenuation,” “Muscular deletion,” “Muscular loss,” “Muscular weak,” “Muscular reduction,” “Muscular strength,” And “Muscular mass.”
